# Psychometric Properties of the Chinese Version of the Oxford Participation and Activities Questionnaire in People with Stroke

**DOI:** 10.3390/ijerph192315450

**Published:** 2022-11-22

**Authors:** Shamay S. M. Ng, Lily Y. W. Ho, Nga-Huen Chan, Tai-Wa Liu, Billy So

**Affiliations:** 1 Department of Rehabilitation Sciences, The Hong Kong Polytechnic University, Hung Hom, Hong Kong SAR, China; 2 Research Centre for Chinese Medicine Innovation, The Hong Kong Polytechnic University, Hung Hom, Hong Kong SAR, China; 3 School of Nursing, The Hong Kong Polytechnic University, Hung Hom, Hong Kong SAR, China; 4 School of Nursing and Health Studies, Hong Kong Metropolitan University, Ho Man Tin, Hong Kong SAR, China

**Keywords:** stroke rehabilitation, activity and participation, psychometric testing, Oxford Participation and Activities Questionnaire

## Abstract

The Oxford Participation and Activities Questionnaire was developed for generic use in the assessment of participation and activity levels. However, it is not available in Chinese and has not been tested in the stroke population. The Oxford Participation and Activities Questionnaire was translated into Chinese and culturally adapted. Its psychometric properties were examined in 100 people with stroke. The participation and activity levels of people with stroke and healthy people were also compared. Content validity and internal consistency (Cronbach’s α = 0.86–0.91) were excellent. The test–retest reliability (intraclass correlation coefficient = 0.91–0.94) was also satisfactory. The standard error of the measurement was 4.10–5.31, and the minimal detectable change was 11.37–14.71. Convergent and divergent validity were supported by hypothesis testing. The instrument had a five-factor structure without a ceiling effect. Its routine activity and social engagement scores discriminated people with stroke from healthy people. In conclusion, the Chinese version of the Oxford Participation and Activities Questionnaire is reliable and valid for assessing participation and activity levels in the stroke population.

## 1. Introduction

The ultimate goal of stroke rehabilitation is to improve independence and reduce the limitations in participation and activities of daily living [1]. Participation is defined as involvement in life situations, and activity is defined as the execution of tasks or actions [2]. After a stroke, almost half of the affected individuals perceive restrictions in performing activities and participating in social roles in their daily living due to long-term impairments [1,3]. Their activity and participation levels improve with time [4], but only approximately 35% of them are able to achieve their pre-stroke levels [5].

Assessing the levels of participation and activity may help to better understand the rehabilitation outcomes and functional recovery of people with stroke. Studies have suggested that improvements in functional status are associated with increased participation and activity in people with stroke (*r* = 0.55) [6]. Moreover, factors associated with participation and activity include psychological well-being [4], physical and motor function [7], and cognitive function [1]. Although a number of instruments have been developed and validated in people with stroke, such as the Stroke Impact Scale, London Handicap Scale, and Assessment of Life Habits, they fail to comprehensively cover both the “activity” and “participation” domains described in the International Classification of Functioning, Disability, and Health (ICF) [8].

The 23-item Oxford Participation and Activities Questionnaire (Ox-PAQ) was developed, based on the ICF model, to assess participation and activity [9,10]. Good to excellent internal consistency (Cronbach’s α = 0.81–0.96) and test–retest reliability (intraclass correlation coefficient (ICC) = 0.83–0.96) have been shown for this instrument in people with motor neuron disease, multiple sclerosis, and Parkinson’s disease [10]. It has also been used to assess people with chronic obstructive pulmonary disease [11]. The instrument has been translated into Turkish [12], but it is not yet available in Chinese and has not been tested in the stroke population.

Clinicians and researchers need an ICF-model-based, psychometrically sound measure to assess participation and activity in people with stroke. Therefore, the objectives of this study were to (i) translate and culturally adapt the Ox-PAQ into Chinese; (ii) examine its content validity, internal consistency, test–retest reliability, ceiling and floor effects, and construct validity; and (iii) compare the levels of participation and activity between people with stroke and healthy people.

## 2. Methods

### 2.1. Translation and Cultural Adaptation

After obtaining permission from the developers, the English version of the Ox-PAQ was translated into Chinese using a protocol recommended by Oxford University Innovation Limited. Two bilingual translators whose mother tongue was Chinese independently translated the Ox-PAQ from English into Chinese. A reconciled translation was then prepared and back-translated from Chinese to English by another two bilingual translators who were not involved in the forward translation.

A panel of five professionals, including two nurses, two physiotherapists, and a professional translator, then evaluated the semantic, experiential, conceptual, and idiomatic equivalence of the initial Chinese version of the Ox-PAQ. A 4-point Likert scale (1 = not relevant; 2 = somewhat relevant; 3 = quite relevant; 4 = highly relevant) was used to assess each item. The back-translated versions were also reviewed by Oxford University Innovation Limited to ensure that the original meaning of the instrument was retained. After pilot testing the instrument on 9 people with stroke, item 16 was culturally adapted by adding “on foot” because not many people in the study region have their own transport. The final Chinese version of the Ox-PAQ (Ox-PAQ-C) was used for psychometric testing.

### 2.2. Setting and Sampling

The stroke participants included in this study were recruited from local self-help groups and non-governmental organizations using convenience sampling. People with stroke were included in the study if they: (1) were aged 50 or above; (2) were able to communicate in Cantonese; (3) were diagnosed with stroke at least 6 months before the start of the study; (4) were community-dwelling; and (5) scored 7 or above in the Abbreviated Mental Test [13]. People with stroke were excluded if they had other neurological diseases. The inclusion and exclusion criteria for healthy participants were the same as those for stroke participants, except that they did not have a diagnosis of stroke.

The lowest ICC previously reported for the Ox-PAQ is 0.83 in people with motor neuron disease, multiple sclerosis, and Parkinson’s disease [10]. The anticipated reliability was set at ICC = 0.80 to estimate the test–retest reliability with sufficient precision. A minimum sample size of 28 was found to be required at 80% power, with a significance level of 0.05. Twenty-nine stroke participants were randomly invited to complete the retest after 7 days. To determine the sample size required for between-group comparison, we used an effect size of 1.05 based on a previous study [14] comparing the activity levels of people with stroke and healthy people. Sample size calculations using G*Power (version 3.1.9.7; Heinrich-Heine-Universität Düsseldorf, Düsseldorf, Germany) showed that at least 16 stroke participants and 16 healthy participants were required, assuming a power of 0.80 and a significance level of 0.05. For exploratory factor analysis (EFA), at least 100 people with stroke would be required [15]. To enhance the robustness of the comparison between the stroke and healthy individual groups, 49 healthy participants were recruited.

### 2.3. Measurements

Oxford Participation and Activities Questionnaire. The Ox-PAQ evaluates participation and activity levels based on the three domains of routine activities, social engagement, and emotional well-being [10]. Each item is measured on a 5-point Likert scale (0 = never; 1 = rarely; 2 = sometimes; 3 = often; 4 = always). The score for each subscale was calculated as $\frac{\mathrm{sum of item scores in the subscale}}{4\;\times \;\mathrm{number of the items in the subscale}}\times$ 100. Higher scores represent greater difficulties with participation and activities [16].

Fugl–Meyer Assessment of the Upper Extremity and Lower Extremity. The Fugl–Meyer Assessment of the Upper Extremity (FMA-UE) and Lower Extremity (FMA-LE) assess reflex actions, synergistic and isolated movements, and coordination of the upper and lower extremities [17]. The FMA-LE uses a 3-point ordinal scale with 17 items, and the FMA-UE consists of 33 items. Higher scores indicate better motor control. Excellent inter-rater reliability has been shown for the FMA-LE (ICC = 0.90) and FMA-UE (ICC = 0.98) in people with stroke [18].

Five Times Sit-to-Stand test. The Five Times Sit-to-Stand (FTSTS) test assesses the strength and transitional movement of the lower limbs [19]. Participants are required to complete five repetitions of a sit-to-stand maneuver as quickly as possible. Three trials were performed by each participant, and the mean completion time was calculated. A shorter completion time indicates better performance. The FTSTS has demonstrated excellent test–retest reliability (ICC = 0.99) in people with stroke [20].

The Chinese version of the Geriatric Depression Scale. The 15-item Chinese version of the Geriatric Depression Scale (CGDS) assesses depressive symptoms [21]. Its total score ranges from 0 to 15, with a higher score indicating more severe depressive symptoms. The CGDS has shown excellent internal consistency (Cronbach’s α = 0.78) in people with stroke [22].

Timed Up and Go test. The Timed Up and Go (TUG) test measures functional mobility. It requires participants to stand up from a standard chair, walk 3 m, turn around, and sit down. Two trials were performed by each participant, and the average time was calculated. A shorter completion time indicates better functional mobility. The test–retest reliability of the TUG test is excellent (ICC = 0.96) in people with chronic stroke [23].

The Chinese version of the Lawton Instrumental Activities of Daily Living Scale. The Chinese version of the Lawton Instrumental Activities of Daily Living Scale (IADL-CV) measures the ability to perform instrumental activities of daily living [24]. It consists of nine items rated on a 3-point ordinal scale, giving a score ranging from 0 to 18. A higher score represents better performance. The IADL-CV has shown good internal consistency (Cronbach’s α = 0.86) and test–retest reliability (ICC = 0.90) in older people [24].

The Chinese version of the Community Integration Measure. The 10-item Chinese version of the Community Integration Measure (CIM-C) captures the perception of community integration [25]. Each item is rated on a 5-point scale, giving a score ranging from 10 to 50, with a higher score indicating better community integration. The CIM-C has demonstrated good internal consistency (Cronbach’s α = 0.84) and test–retest reliability (ICC = 0.84) in people with stroke [25].

### 2.4. Statistical Analysis

The Statistical Package for the Social Sciences (version 26.0; IBM, Armonk, NY, USA) was used to analyze quantitative data. Missing variables were excluded in a pairwise manner. The demographic characteristics of the stroke participants and healthy participants were summarized with descriptive statistics and compared using an independent Student’s *t*-test or chi-square test. The item-level content validity index (I-CVI) and scale-level content validity index (S-CVI) values were calculated. An I-CVI value ≥0.78 and an S-CVI value ≥0.9 were considered as indicating excellent content validity [26]. Internal consistency was considered to be acceptable when Cronbach’s α values ranged from 0.70 to 0.95 [27].

Test–retest reliability was established using ICC_3,1_. ICC values <0.50, 0.50–0.75, 0.75–0.90, and >0.90 were used to indicate poor, moderate, good, and excellent reliability, respectively [28]. The standard error of measurement (SEM) was calculated as $S\times \sqrt{1-r}$, and the minimal detectable change (MDC) was calculated as SEM $\times \sqrt{2}\times 1.96$, where S is the standard deviation of the scores at baseline and r is the ICC coefficient. More than 15% of the participants achieved the highest or lowest scores on the Ox-PAQ-C, indicating that ceiling and floor effects occurred [29].

The construct validity of the Ox-PAQ-C was assessed by EFA using principal component analysis with varimax rotation. The number of factors was defined using a scree plot. Convergent and divergent validity were investigated by hypothesis testing. For convergent validity, we hypothesized moderate to strong correlations between the routine activities subscale and the CIM and IADL-CV and between the social engagement subscale and the CIM, as they measured activity and participation domains in the ICF model. Moreover, a moderate to strong correlation was expected between the emotional well-being subscale scores and CGDS scores because the CGDS assesses depressive symptoms, which are related to the emotional well-being of the respondents. In addition, divergent validity was assessed by hypothesizing weak correlations between the Ox-PAQ-C scores and the FMA-LE, FMA-UE, FTSTS, and TUG test, as they measure physical function but not the levels of activity and participation. Spearman’s correlation coefficient (*r_s_*) was calculated, and values of 0.10–0.39, 0.40–0.69, 0.70–0.89, and >0.90 were defined as weak, moderate, strong, and very strong correlations, respectively [30].

### 2.5. Ethical Considerations

The guidelines of the Declaration of Helsinki were followed in this study. Ethical approval was granted by The Hong Kong Polytechnic University. Written informed consent was obtained from all participants.

## 3. Results

### 3.1. Demographic Characteristics

In total, 100 people with stroke and 49 healthy people were recruited for this study. Their demographic characteristics are summarized in Table 1. Missing data in both the FMA-UE and TUG tests were found for one participant with stroke. Missing data for the FTSTS test were found for four participants with stroke.

### 3.2. Content Validity

The I-CVI values of all items were 1.00, and the S-CVI value was 1.00.

### 3.3. Internal Consistency

The Cronbach’s α values for routine activities, social engagement, and emotional well-being scores were 0.91, 0.86, and 0.91, respectively. The corrected item-total score correlation is shown in Table 2.

### 3.4. Test–Retest Reliability

The ICC, SEM, and MDC values were from 0.91 to 0.94, from 4.10 to 5.31, and from 11.37 to 14.71, respectively (Table 3).

### 3.5. Ceiling and Floor Effects

None of the participants obtained the highest routine activities or social engagement scores, and only one participant (1%) obtained the highest score for emotional well-being. However, 16 (16%) participants obtained the lowest routine activities score, 40 (40%) obtained the lowest social engagement score, and 33 (33%) obtained the lowest emotional well-being score.

### 3.6. Convergent and Divergent Validity

As expected, convergent validity was supported by significant moderate correlations between routine activities scores and CIM (*r_s_* = −0.405) and IADL-CV (*r_s_* = −0.595) scores and between emotional well-being and CGDS scores (*r_s_* = 0.404). However, the correlations between social engagement and CIM scores were weak (*r_s_* = −0.351) and did not support the hypothesis regarding convergent validity (Table 4).

Weak correlations of the three subscales of the Ox-PAQ-C with the FMA-LE (*r_s_* = −0.288 to −0.055) and FMA-UE (*r_s_* = −0.174 to −0.010) supported the hypotheses regarding divergent validity. However, significant moderate correlations between routine activities scores and FTSTS (*r_s_* = 0.404) and TUG times (*r_s_* = 0.406) did not support the hypotheses regarding divergent validity (Table 4).

### 3.7. Construct Validity

Bartlett’s test of sphericity (*p* < 0.001) showed a satisfactory factor analysis [31]. The Kaiser–Meyer–Olkin coefficient was 0.869, indicating sampling adequacy for the factor analysis [31]. The EFA suggested a five-factor structure that included “social engagement and life activities”, “emotional well-being”, “daily activities and transportation”, “routine activities”, and “community engagement” and explained 72% of the total variance (Table 5).

### 3.8. Comparison between People with Stroke and Healthy People

Stroke participants had significantly higher scores than healthy participants for the subscales of routine activities and social engagement but not for the subscale of emotional well-being (Table 6).

## 4. Discussion

This was the first study to translate the Ox-PAQ into Chinese and investigate its psychometric properties in people with stroke. The Ox-PAQ-C showed excellent content validity, internal consistency, and test–retest reliability. A five-factor structure was found in people with stroke. The stroke participants had significantly higher scores than healthy participants for routine activities and social engagement.

In the present study, we established the equivalence between the English and Chinese versions of the Ox-PAQ-C using an expert panel of five professionals with different training backgrounds. This may greatly contribute to the assessment of the levels of participation and activity in the Chinese stroke population. The Cronbach’s α value obtained for the Ox-PAQ-C was comparable to the value obtained for the original Ox-PAQ (Cronbach’s α = 0.81–0.96) [10], indicating the consistency of the Ox-PAQ items for assessing the constructs of participation and activity in Western and Chinese societies.

The Ox-PAQ-C demonstrated excellent test–retest reliability, with ICC values similar to those of the original Ox-PAQ (ICC = 0.83–0.96) [10] and the Turkish version of the Ox-PAQ (ICC = 0.96–0.98) [12]. These findings suggest that the Ox-PAQ-C precisely conveyed the same meaning and yielded consistent responses from participants of different cultures.

No previous study has explored the SEM and MDC of the Ox-PAQ. The MDC values for all subscales in this study were slightly greater than the acceptable range, which was 10% [32]. One possible reason contributing to such findings in the present study may be the inconsistent activities reported by respondents. For example, the participants may remember different activities between the test and retest, resulting in inconsistent responses to item 6.

In contrast to the Turkish version of the Ox-PAQ, which showed no ceiling or floor effects [12], floor effects were found for all subscales of the Ox-PAQ-C. As the participants of the present study were a cohort of community-dwelling people with chronic stroke, it is possible that they may have developed their own compensatory strategies after their stroke to overcome difficulties in participation and activity. They may perceive only minimal difficulties in participation and activities, and thus, most of the stroke participants chose the lowest-scoring response on the Ox-PAQ items.

The convergent validation results in this study supported our stated hypotheses and suggest that the subscales of the Ox-PAQ-C measure concepts similar to those measured by the CIM-C, IADL-CV, and CGDS. The items in the routine activities subscale included activities of daily living and community participation, which are linked to the constructs of the CIM-C and IADL-CV. The emotional well-being subscale included items assessing the negative emotions of the respondents, which were similar to the items used to indicate depressive traits in the CGDS. However, only a weak correlation was found between the social engagement score and the CIM-C score, which did not support our hypothesis. A possible explanation is that the items in the social engagement subscale of the Ox-PAQ-C mainly focus on communication and relationships with others, while the CIM-C comprises more domains, such as “independent living” and “sense of knowing” [25]. Thus, the correlation between the two measures was weak in this study.

Surprisingly, although the construct of the Ox-PAQ-C was different from the constructs of tests assessing the strength and transitional movement of the lower limbs and functional mobility, divergent validity between the routine activities subscale scores and the FTSTS and TUG times were not supported in this study. This may be because routine activities require rapid rates of muscle contraction in the lower limbs [33]. Muscle strength and functional mobility may also influence the routine performance of dynamic lower limb activities in people with stroke [33,34], resulting in moderate correlations between the routine activities subscale and the FTSTS and TUG times.

The EFA revealed a five-factor structure in the Ox-PAQ-C, which was different from the three-factor structure in the original Ox-PAQ [10] and the Turkish version of the Ox-PAQ [12]. Such discrepancies may be explained by differences in sample characteristics. Our study included stroke participants, whereas individuals with motor neuron disease, multiple sclerosis, and Parkinson’s disease were recruited in the study of the original Ox-PAQ [10], and healthy older people were included in the study of the Turkish version of the Ox-PAQ [12]. Impairments experienced by different population groups are not the same, resulting in different perceived limitations in routine activities and social engagement. However, the factor of emotional well-being was consistent across all language versions because all items under this factor were clearly related to emotion.

The Ox-PAQ-C routine activities and social engagement subscales significantly discriminated between people with stroke and healthy people. Impairments in physical ability after a stroke lead to difficulties in the activities of daily living and participation in social activities [35]. However, the emotional well-being score showed no significant difference between the two groups. Although disability may negatively affect the emotional well-being of people with stroke [36], the ability to regulate emotions tends to improve over time because people with chronic stroke may learn to adapt and modulate their emotions [37]. Most of our stroke participants had a long post-stroke duration, with an average greater than 7 years, and actively participated in self-help groups. Thus, they were more likely to have a positive emotional state, resulting in no significant difference in emotional well-being scores when compared with healthy people.

This study has some limitations. The participants in this study were recruited from local self-help groups, and thus, they may have higher levels of participation and activity than other people with stroke. Therefore, the generalizability of our findings may be limited. Additionally, the retest interval in this study was 7 days, which might not be sufficient to eliminate recall bias. Thus, the results in test–retest reliability should be interpreted with caution. Moreover, the sample size in this study was barely adequate for EFA. A larger sample size should be considered in future studies.

## 5. Implications

Healthcare professionals can adopt the Ox-PAQ-C to assess levels of participation and activity and to evaluate the effectiveness of interventions in people with stroke. In future studies, testing the Ox-PAQ-C in different populations is recommended to explore different constructs of the instrument. This may facilitate an understanding of activity limitations and participation restrictions for different populations. Future studies may be conducted to confirm the SEM and MDC values.

## 6. Conclusions

The Ox-PAQ-C is a reliable and valid instrument to assess the levels of participation and activity in community-dwelling people with stroke. Its routine activities and social engagement scores discriminate people with stroke from healthy people. The level of participation and activity can be considered as one of the outcomes in stroke rehabilitation to evaluate and compare the effects of different interventions.

## Figures and Tables

**Table 1:** Demographic characteristics of the participants.

Characteristics	Stroke (*n* = 100)	Healthy (*n* = 49)	*p*-Value
Age, mean (SD)	65.00 (6.18)	62.90 (7.29)	0.068
Gender, number (%)			
Male	58 (58.00)	14 (28.57)	0.001
Female	42 (42.00)	35 (71.43)	
Years since stroke, mean (SD)	7.76 (4.44)	/	
Marital status, number (%)			0.034
Single	9 (9.00)	7 (14.29)	
Married	75 (75.00)	41 (83.67)	
Divorced/widowed	16 (16.00)	1 (2.04)	
Use of walking aid, number (%)			<0.001
No	28 (28.00)	49 (100.00)	
Yes	72 (72.00)	0 (0.00)	
Number of household members, mean (SD)	2.79 (1.06)	2.90 (1.33)	0.592

SD, standard deviation.

**Table 2:** Internal consistency of the Chinese version of the Oxford Participation and Activities Questionnaire.

	Cronbach’s Alpha	Corrected Item-Total Correlation	Alpha If Item Deleted
*Routine activities*	0.91		
1. Getting up in the morning		0.51	0.91
2. Getting dressed		0.63	0.90
3. Getting around home		0.52	0.91
4. Doing household chores		0.63	0.90
5. Going to shops		0.65	0.90
6. Daily activities you like to do		0.53	0.91
7. Doing work, paid or unpaid		0.63	0.90
8. Social life		0.70	0.90
9. Leisure activities		0.65	0.90
10. Physical activities for enjoyment		0.77	0.90
14. Being as independent as would like		0.58	0.91
15. Engaging in community life		0.60	0.90
16. Using own transport		0.61	0.91
17. Using public transport		0.70	0.90
*Social engagement*	0.86		
11. Maintaining close relationships		0.78	0.78
12. Maintaining friendships		0.77	0.79
13. Engaging in the community		0.63	0.85
18. Communicating with others		0.63	0.85
*Emotional well-being*	0.91		
19. Control over life		0.63	0.92
20. Stressed		0.78	0.89
21. Anxious		0.85	0.87
22. Sad		0.84	0.88
23. Depressed		0.79	0.89

**Table 3:** Test–retest reliability, standard error of measurement, and minimal detectable change of the Chinese version of the Oxford Participation and Activities Questionnaire.

	Intraclass Correlation Coefficients (95% Confidence Interval)	Standard Error of Measurement	Minimal Detectable Change
Routine activities	0.94 (0.87–0.97)	4.10	11.37
Social engagement	0.91 (0.82–0.96)	5.31	14.71
Emotional well-being	0.92 (0.83–0.96)	5.16	14.31

**Table 4:** Convergent and divergent validity of the Chinese version of the Oxford Participation and Activities Questionnaire.

Variables	Routine Activities		Social Engagement		Emotional Well-Being	
	*r_s_*	*p*	*r_s_*	*p*	*r_s_*	*p*
*Convergent validity*						
Geriatric Depression Scale	0.343 **	<0.001	0.232 *	0.020	0.404 **	<0.001
Community Integration Measure	−0.405 **	<0.001	−0.351 **	<0.001	−0.332 **	0.001
Lawton Instrumental Activities of Daily Living Scale	−0.595 **	<0.001	−0.391 **	<0.001	−0.263 **	0.008
*Divergent validity*						
Fugl-Meyer Assessment for the lower extremity	−0.288 **	0.004	−0.104	0.303	−0.055	0.588
Fugl-Meyer Assessment for the upper extremity	−0.174	0.086	−0.010	0.921	−0.131	0.195
Five Time Sit-To-Stand test	0.404 **	<0.001	0.199	0.052	0.179	0.082
Timed Up and Go test	0.406 **	<0.001	0.141	0.164	0.109	0.282

* *p* < 0.05. ** *p* < 0.01. Notes. Expected convergent validity is in bold. Expected divergent validity is in italics. Boxed values indicate that the hypotheses are supported.

**Table 5:** Rotated factor matrix of the Chinese version of Oxford Participation and Activities Questionnaire based on the principal component analysis with varimax rotation.

Item	Factor				
	1	2	3	4	5
13. Engaging in the community	0.81				
12. Maintaining friendships	0.80				
11. Maintaining close relationships	0.76				
9. Leisure activities	0.76				
8. Social life	0.59				
6. Daily activities you like to do	0.58				
10. Physical activities for enjoyment	0.53				
22. Sad		0.91			
23. Depressed		0.88			
21. Anxious		0.84			
20. Stressed		0.78			
19. Control over life		0.51			
5. Going to shops			0.80		
17. Using public transport			0.77		
16. Using own transport			0.75		
4. Doing household chores			0.67		
3. Getting around home				0.81	
2. Getting dressed				0.68	
7. Doing work, paid or unpaid				0.66	
1. Getting up in the morning				0.64	
15. Engaging in community life					0.72
14. Being as independent as would like					0.67
18. Communicating with others					0.63
Eigenvalues	9.89	2.43	1.87	1.38	1.00
Variance explained (%)	43.02	10.60	8.12	6.01	4.35

**Table 6:** Comparisons of the Oxford Participation and Activities Questionnaire scores between the people with stroke and healthy people.

Subscale	Median (Interquartile Range)		Mann–Whitney U	Z	*p*
	Stroke (*n* = 100)	Healthy (*n* = 49)			
Routine activities	14.26 (29.92)	0.00 (8.93)	1244.50	−4.93	<0.001 **
Social engagement	6.25 (31.25)	0.00 (12.50)	1763.50	−2.94	0.003 **
Emotional well-being	15.00 (35.00)	10.00 (22.50)	2070.50	−1.58	0.115

** *p* < 0.01.

## Data Availability

The data presented in this study are available on request from the corresponding author. The data are not publicly available due to privacy issues of the participants.
